# *Lactobacillus paracasei* ET-22 Suppresses Dental Caries by Regulating Microbiota of Dental Plaques and Inhibiting Biofilm Formation

**DOI:** 10.3390/nu15153316

**Published:** 2023-07-26

**Authors:** Meng Guo, Jianmin Wu, Weilian Hung, Zhe Sun, Wen Zhao, Hanglian Lan, Zhi Zhao, Guna Wuri, Bing Fang, Liang Zhao, Ming Zhang

**Affiliations:** 1 School of Food and Health, Beijing Technology and Business University, Beijing 100024, China; guomeng_elena@163.com (M.G.);; 2 Key Laboratory of Precision Nutrition and Food Quality, Department of Nutrition and Health, China Agricultural University, Beijing 100083, China; 3 Inner Mongolia Dairy Technology Research Institute Co., Ltd., Hohhot 010110, China; 4 National Center of Technology Innovation for Dairy, Hohhot 010110, China; 5 Key Laboratory of Functional Dairy, College of Food Science and Nutritional Engineering, China Agricultural University, Beijing 100083, China; 6 Beijing Laboratory of Food Quality and Safety, College of Food Science and Nutritional Engineering, China Agricultural University, Beijing 100083, China

**Keywords:** *L. paracasei*, dental caries, biofilm, dental plaques

## Abstract

Dental caries is a common and multifactorial biofilm disease that is associated with dietary habits and microbiota. Among the various pathogens inducing caries, *S. mutans* is the most extensively studied. Promoting oral health with probiotics has gained considerable attention. *Lactobacillus paracasei* (*L. paracasei*) strains were reported to modulate the gut microbiota and enhance host resistance to disease. Our previous research has found that *L. paracasei* ET-22 (ET-22) could inhibit *S. mutans* biofilms in vitro. However, the preventive effect in vivo and functional mechanism of ET-22 on dental caries were unclear. In this study, the preventive effects of ET-22 on dental caries in mice were checked. Meanwhile, the functional mechanism of ET-22 was further investigated. Results showed that the supplementation of ET-22 in drinking water significantly improved the caries scoring of mice. The microbiota of dental plaques revealed that the live and heat-killed ET-22 similarly regulated the microbial structure in plaque biofilms. Functional prediction of PICRUSt showed that the addition of live and heat-killed ET-22 may inhibit biofilm formation. By the in vitro trials, the live and heat-killed ET-22 indeed inhibited the construction of *S. mutans* biofilms and EPS productions of biofilms. This evidence suggests that ET-22 can restrain dental caries by regulating the microbiota of dental plaques and inhibiting biofilm formation, which may be partly mediated by the body components of ET-22.

## 1. Introduction

Dental caries remains the most common chronic infectious disease, leading to the progressive destruction of teeth and bringing huge burdens on the public health prevention system [1,2]. Based on the estimates of the World Health Organization (WHO), untreated dental caries in permanent teeth is the most widespread health condition. Most specifically, 2.3 billion people and more than 530 million children suffer from caries of permanent teeth and primary teeth, respectively [3,4]. Dental plaque, a bacterial biofilm that adheres to the surface of teeth, is an important cause of tooth defects and caries. Currently, dental plaque is mainly prevented and controlled by using public health interventions, e.g., well-balanced and low or sugar-free diets, adequate exposure to fluoride, mechanical removal techniques by daily teeth brushing and flossing, and antimicrobial agents [4,5].

Accumulated evidence indicates that host–microbial interactive networks in the oral cavity are coordinated and work by an ecological chronometer of health conditions, playing key effects on dental and periodontal diseases [6,7]. As a polymicrobial biofilm disease, caries is driven by the host factors, such as dietary sugars, and microbiota–matrix interactions that happen on dental surfaces [8]. Sugars can aggravate the proliferation of some pathogens, assembly of the matrix, and acidification of the microenvironment inside biofilms, which promotes ecological changes and concerts multispecies efforts to damage the mineralized tooth surface [9]. Furthermore, the dental caries etiology and pathogenesis highlight that the ‘mixed bacterial-ecological approach’ assumes the responsibility for the occurrence and development of lesions [10,11]. When the *Streptococcus mutans* (*S. mutans*), the most notorious oral pathogen, obtain an advantage over others, a calamitous ecological transition inside the plaque biofilms occurs and ultimately tips the caries process. Together, inhibiting the formation of cariogenic biofilms and maintaining a balanced oral microbiota are the essential approaches for caries prevention.

Some views have emerged that probiotics are a new method getting acceptance to maintain the health of dental tissues [12,13]. Interestingly, *Lactobacillus* interventions show positive impacts on oral health in studies for many years, in which beneficial effects of *Lactobacillus* species on *S. mutans* most likely result from the capacity of inhibiting their growth, biofilm formation, and functional gene expressions [14]. Some biofilm-forming bacteria treated with the supernatants of *Lactobacillus*, such as the *Lactobacillus salivarius* (*L. salivarius*), *L. casei*, *L. reuteri*, and *L. plantarum*, showed the reduced genes expressions involved in exopolysaccharide (EPS) production, acid tolerance, and quorum sensing [15,16]. Also of interest is the fact that not only live lactobacilli but also paraprobiotics have been regarded as an adjunctive therapy for the treatment of oral disease [17]. Although some *Lactobacillus* strains present direct antagonism and competitiveness against specific pathogens, the regulated mechanisms inside dental plaques are poorly understood, which highlights the significance to investigate the biological prevention measures for dental caries [18,19,20,21].

Some strains of *L. paracei* have shown beneficial effects on the oral cavity [12]. The *L. paracasei* ET-22 (ET-22) was acquired from the intestines of a healthy infant and viewed as a safe probiotic [22]. Meanwhile, our in vitro trials have found ET-22 could inhibit the *S. mutans* biofilms [23]. In this study, we first made a mouse model with dental caries with a high sucrose diet and then researched the regulatory roles of ET-22 on the microbiota inside dental plaques. In addition, the effects of live and heat-killed ET-22 on the growth and biofilm construction of *S. mutans* on saliva-coated hydroxyapatites were tested in vitro trials. This was the first evaluation of the ET-22 on dental caries and revealed a promising new strategy to prevent or improve dental caries induced by *S. mutans*.

## 2. Materials and Methods

### 2.1. Bacterial Strains and Cultivation

Sanhe Fucheng Biotechnology Co., Ltd. (Hebei, Sanhe, China) helped to provide the ET-22 (CGMCC No. 15077). The pathogen *S. mutans* (CGMCC 1.2499) was purchased from the China General Microbiological Culture Collection Center (Beijing, China). The commercial MRS and BHI mediums were used to for cultivation of ET-22 and *S. mutans*, respectively.

### 2.2. Preparing Bacteria Samples

The ET-22 and *S. mutans* were cultured at 37 °C for about 12 h and then collected by centrifugation at 4000× *g* for 10 min. After washing, the concentration of bacteria was adjusted to 5 × 10^8^ CFU/mL by measuring OD600 absorbance and serial dilutions and growth on MRS culture plates. The same dose of bacteria in PBS was used to prepare the live and heat-killed ET-22. The heat-killed ET-22 was prepared by heating at 70 °C for 1 h [23]. The live and heat-killed ET-22 were made into lyophilized powder with a lyophilizer. All bacterial samples were freshly prepared before the trials.

### 2.3. The Animal Model of Dental Caries and Treatments with Live and Heat-Killed ET-22

**Figure 1:**
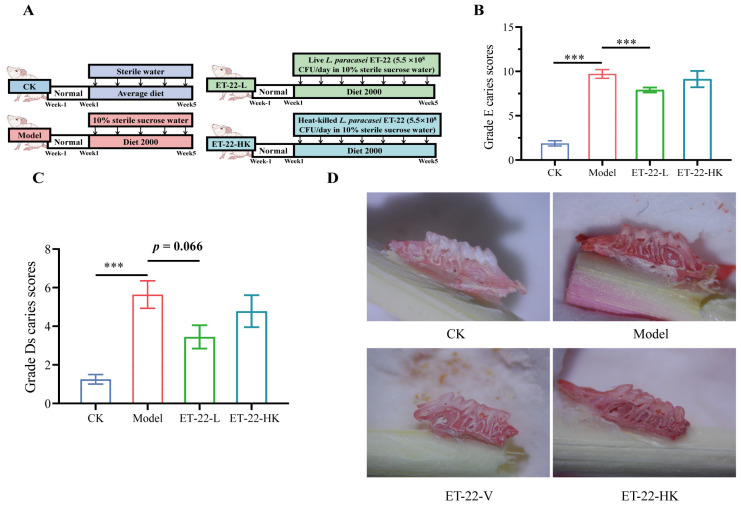
Effects of ET-22 on dental caries of mice. (**A**) Experiment arrangements, including water, diet, and liver or heat-killed ET-22. (**B**) Grade E caries scores for enamel carious damages of mice. (**C**) Grade D caries scores for severities of dentin carious lesions of mice. (**D**) The representative molar images of jawbones. CK, the mice treated by the sterile water and average diet; Model, the mice treated by the 10% sterile sucrose water and diet 2000; ET-22-L, the mice treated by live ET-22 in 10% sterile sucrose water and diet 2000; ET-22-HK, the mice treated by heat-killed ET-22 in 10% sterile sucrose water and diet 2000. Mean ± SEM is shown (*n* = 10). 0.05 ≤ *p* < 0.1 meant a trend to show significant difference; *** *p* < 0.001.

This animal experiment was approved by the China Agricultural University Institutional Animal Care Committee (No. AW01210202-4-3) and conducted in line with the guidelines for the care and use of laboratory animals of China Agricultural University. Based on a widely used mouse model of dental caries, the prevention effects and mechanisms of ET-22 were investigated. The four experimental diets (Figure 1A) for this trial were prepared based on the methods described previously [24,25]. A total of 40 6-week-old male SPF BALB/c mice weighing 22.3 ± 1.8 g were purchased from Vital River Laboratory Animal Technology Co., Ltd. (Beijing, China) and were. randomly divided into 4 groups with 10 each, including the CK, Model, ET-22-L, and ET-22-HK, respectively. The 10 mice in each group were kept in 5 cages. The mice of CK group or other groups were fed with the regular diet or cariogenic diet with 56% sucrose (Diet 2000; Trophic Animal Feed High-Tech Co., Ltd., Nantong, China). Meanwhile, they were provided sterile water or 10% (*w*/*v*) sterile sucrose water. In the first 5 days, mice of CK group or other groups were daily inoculated with 50 µL vehicle or live *S. mutans*. The vehicle was 1.2% (*w*/*v*) carboxymethylcellulose in PBS. The inoculations were finished by a swab stick to rotate onto the teeth at 9 am. In addition, ET-22 cells were supplemented into the drinking water of ET-22-L and ET-22-HK groups once per day, which could keep the probiotics in mouth approximately from 5 p.m. to 9 a.m. [26]. The doses of ET-22 and pathogenic *S. mutans* for each mouse were all settled to 5.0 × 10^8^ CFU/day. However, for the CK and Model groups, the supplemented substances were replaced with the same volume of PBS. This trial duration was 5 weeks, during which the water and feed consumption were controlled to ensure all groups received same volume and weight of water and feed. All mice were kept in SPF house with the 23 ± 2 °C and 50–60% relative humidity. At the end of the study, all mice in each group were euthanized by the isoflurane asphyxiation and sacrificed by the cervical dislocation. The teeth tissues were collected.

### 2.4. Caries Scoring of Mice

After being sacrificed, the heads of mice were collected to boil for 15 min. Then, all mouth cavity samples were surgically dissected, and the jawbones were obtained. A total of 0.4% (*m*/*v*) murexide (Sigma-Aldrich, St. Louis, MO, USA) was used to stain such jawbones for 12 h. After being rinsed, the samples were semi-sectioned along the occlusal surfaces between the maxillary and mandibular molars. Subsequently, under a stereomicroscope, the molars were observed and estimated by a standard method, the reported Keyes caries scoring system, in which the linear evaluation of enamel carious damages was performed by the grade E caries scoring method, while the levels of dentin carious lesions were evaluated by the grade D caries scoring method [24,27,28]. A group of inspectors who were blind for the study performed this caries scoring.

### 2.5. Collecting Dental Plaques

To assess the microbial community structure, samples of supragingival dental plaques were collected from available exposed tooth surfaces during the last 6 days by the sterile Gracey curettes (Hu-Friedy, Shanghai, China). Following this method, pooling the 6 day microbial samples for each mouse was used to evaluate the bacterial diversity and community abundances [29]. Pooled plaque samples were saved in ultra-low temperature storage freezer until DNA extraction.

### 2.6. 16S rRNA Gene Sequencing of Dental Plaques

Genomic DNA from pooled plaque samples was extracted with the commercial MP FastDNATM SPIN Kits (MP Biomedicals, Santa Ana, CA, USA). The third generation of sequencing technology was used to read the full regions of 16S rRNA genes to more accurately identify the changed species of microbiota. The full length of the bacterial 16S rRNA genes was amplified with a pair of universal primer 27F (5′-AGRGTTYGATYMTGGCTCAG-3′) and 1492R (5′-RGYTACCTTGTTACGACTT-3′) tagged with barcodes by a GeneAmp^®^ 9700 PCR thermocycler (ABI, Tampa, FL, USA) [30]. After the agarose gel electrophoresis, the PCR products with about 1500 bp were recovered with the commercial AMPure^®^ PB beads (Pacifc Biosciences, Menlo Park, CA, USA) and subsequently checked the concentrations with the Quantu^TM^ Fluorometer (Promega, Madison, WI, USA). After pooling purified products in proportion to the required volume for each sample, the DNA libraries were established with the SMRTbell^®^ Express Template Prep Kit 2.0 (Pacifc Biosciences, USA), following the instruction provided. Sequences of SMRTbell libraries were read via the Pacbio Sequel II System (Pacific Biosciences, USA) with the help of Shanghai Majorbio Bio-pharm Technology Co., Ltd. (Shanghai, China).

SMRTLink analysis software (version 8.0) was used to process the raw data to obtain demultiplexed circular consensus sequence (CCS) reads. After barcode-identifying and length-filtering, the rRNA gene sequences with lengths between 1000 and 1800 bp were retained. According to the 97% sequence similarity by UPARSE 7.1, the optimized CCS reads were clustered into operational taxonomic units (OTUs) [31,32]. To control effects of discrepant sequencing depths on diversity measurements, the sequence number for each sample was uniformly quantified to 5202, which covered 99% of sequences. Representative sequences from OTUs were compared with the Silva 138 database to annotate the species information by the RDP Classifier 2.2 based on the confidence threshold of 0.7 [33]. On the basis of the OTUs information, the alpha diversity including Sobs and Shannon indexes, was calculated with the Mothur 1.30.1 [34]. Principal coordinate analysis (PCoA) and visualization reflected by the Bray-Curtis distances were used to present the beta diversity of microbial communities. Another analysis of the dental plaque microbiota was finished with the help of Majorbio Cloud platform (https://cloud.majorbio.com, accessed on 23 March 2023) [35]. At different taxonomy levels, the relative abundances of communities were plotted by the GraphPad Prism software (9.0.0 edition).

### 2.7. In Vitro Biofilm Model and Treatments with Live and Heat-Killed ET-22

The construction method of in vitro biofilm model followed previous research [22]. Briefly, sucrose and hydroxyapatite discs were supplemented into the artificial-saliva medium, and then the medium was infused into sterile 24-well culture plates. Pathogenic *S. mutans* was added to achieve 5 × 10^8^ CFU/mL. Under the condition of anaerobic culture with the help of anaerobic-culture bags and AnaeroPacks (Mitsubishi Gas Chemical Company, Inc., Tokyo, Japan) for 1 day, the *S. mutans* biofilms formed. In order to make clear the functions of live and heat-killed ET-22 on the *S. mutans* biofilms, they were co-cultured with *S. mutans*. For each treatment, 50 µL PBS or live or dead cells were, respectively added to co-culture with the *S. mutans*. One day later, the *S. mutans* biofilms were elevated in the growth degree. Scanning electron microscope was applied to check the structural changes of *S. mutans* biofilms. At the same time, the Alexa Fluor™ 647 (Thermo, Waltham, MA, USA) dye was used to stain the live bacteria and showed red color. And the SYTO™ 9 (Thermo, USA) dye was used to stain both live and dead bacteria and showed green color. Staining results were recorded with confocal laser scanning microscopy (Leica, Wetzlar, Germany). By two-dimensional (2D) and three-dimensional (3D) patterns, the results were recorded, respectively. Furthermore, the soluble and insoluble extracellular polysaccharide (EPS) were detected to elevate the biofilms formation. The detection methods followed a previous article [36].

### 2.8. Statistical Analysis

All data were shown as Means with SEM. We used SPSS 26.0 software for the statistical analyses in this experiment. When the variance was even, the student’s t-test was used for comparison among groups. Otherwise, the Mann–Whitney test was used. For the multiple groups, one-way ANOVA and LSD post hoc test or Tamhane’s T2 test was used to compare the data. For the functional predictions of microbiota, we used Kruskal–Wallis H test and Wilcoxon rank-sum test to compare the differences. 0.05 ≤ *p* < 0.1 showed a trend to difference. When *p* < 0.05, the difference was significant. * *p* < 0.05, ** *p* < 0.01, *** *p* < 0.001. The results were plotted by the Majorbio Cloud platform (https://cloud.majorbio.com, accessed on 26 March 2023) and GraphPad Prism software (9.0.0 edition).

## 3. Results

### 3.1. Effects of ET-22 on Dental Caries

In order to check the impacts of probiotics cells on dental caries, live and heat-killed ET-22 were supplemented into the drinking water of mice. And the grade E caries scoring and grade Ds caries scoring methods were, respectively used to evaluate the enamel damages and severities of carious lesions. Results showed that compared with the CK group, the Model group showed a significantly increased grade E caries score and grade Ds caries score (*p* < 0.001, Figure 1B,C). In the comparisons between the Model group with the other two groups, the ET-22-L group showed an obviously decreased grade E caries score (*p* < 0.05, Figure 1B) and a reduced trend of grade Ds caries score (0.05 < *p* < 0.1, Figure 1C). However, the ET-22-HK group did not show significant differences in two caries scores compared to the Model group (*p* > 0.05, Figure 1B,C). Moreover, the representative images from these four groups were presented in Figure 1D.

### 3.2. Effects of ET-22 on the Diversity of Microbiota in Dental Plaques

**Figure 2:**
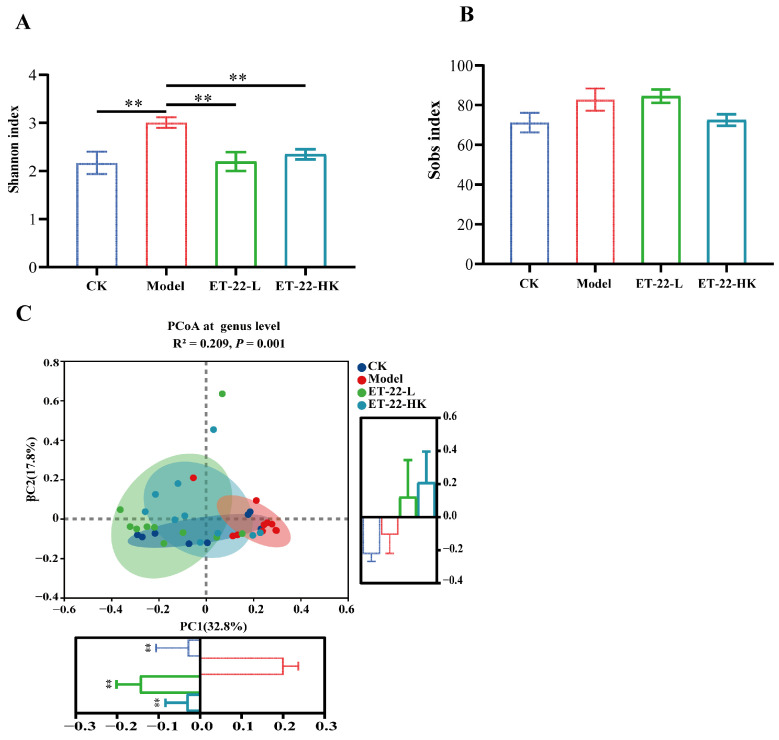
Effects of ET-22 on the diversity of microbiota in dental plaques (**A**,**B**). The alpha diversity of microbiota in dental plaques was evaluated by the Shannon index (**A**) and Sobs index (**B**) based on the clustered OTUs. (**C**) The beta diversity of microbiota in dental plaques was evaluated by the PCoA analysis at the genus level based on the bray–curtis distances. For the PC1 and PC2, the values of groups were compared, respectively. CK, the mice treated by the sterile water and average diet; Model, the mice treated by the 10% sterile sucrose water and diet 2000; ET-22-L, the mice treated by live ET-22 in 10% sterile sucrose water and diet 2000; ET-22-HK, the mice treated by heat-killed ET-22 in 10% sterile sucrose water and diet 2000. Mean ± SEM are shown (*n* = 10). ** *p* < 0.01, vs. Model group.

In order to investigate the effects of ET-22 on subgingival plaque biofilms, we examined the microbiota of supragingival dental plaques by the full-length sequencing of 16S rRNA genes, which can accurately identify bacterial communities at the species level. Based on the clustered OTUs, the alpha diversity evaluated by the Shannon and Sobs indexes was analyzed. As can be seen in Figure 2A, after being co-treated with the cariogenic feed and *S. mutans* infection, the Model group showed a significantly raised Shannon index compared to the CK group (*p* < 0.05). However, the ET-22-L and ET-22-HK treatments significantly decreased the Shannon index (*p* < 0.05, Figure 2A). Different from the Shannon index, there was no statistical difference in the Sobs index among these groups (*p* > 0.05, Figure 2B).

To overview the effects of ET-22 on the landscape of microbiota in dental plaques, the beta diversity reflected by the PCoA based on the Bray–Curtis distances was performed. According to the visualization result of PCoA, the variation values of PC1 and PC2 are 26.52% and 16.77%, respectively (Figure 2C). Obviously, the samples of Model group gathered and are relatively independent from those of other groups (Figure 2C). There was minor overlap between the CK group and model group (Figure 2C). Despite similarly less superposition, the ET-22-L and ET-22-HK groups had greater overlap and clearly segregated from the Model group (Figure 2C). For the PC1, the CK, ET-22-L, and ET-22-HK groups were all significantly lower than Model group (*p* < 0.05, Figure 2C). As for the PC2, there was no obvious difference among these four groups.

### 3.3. Influences of ET-22 on the Structure of Microbiota in Dental Plaques

**Figure 3:**
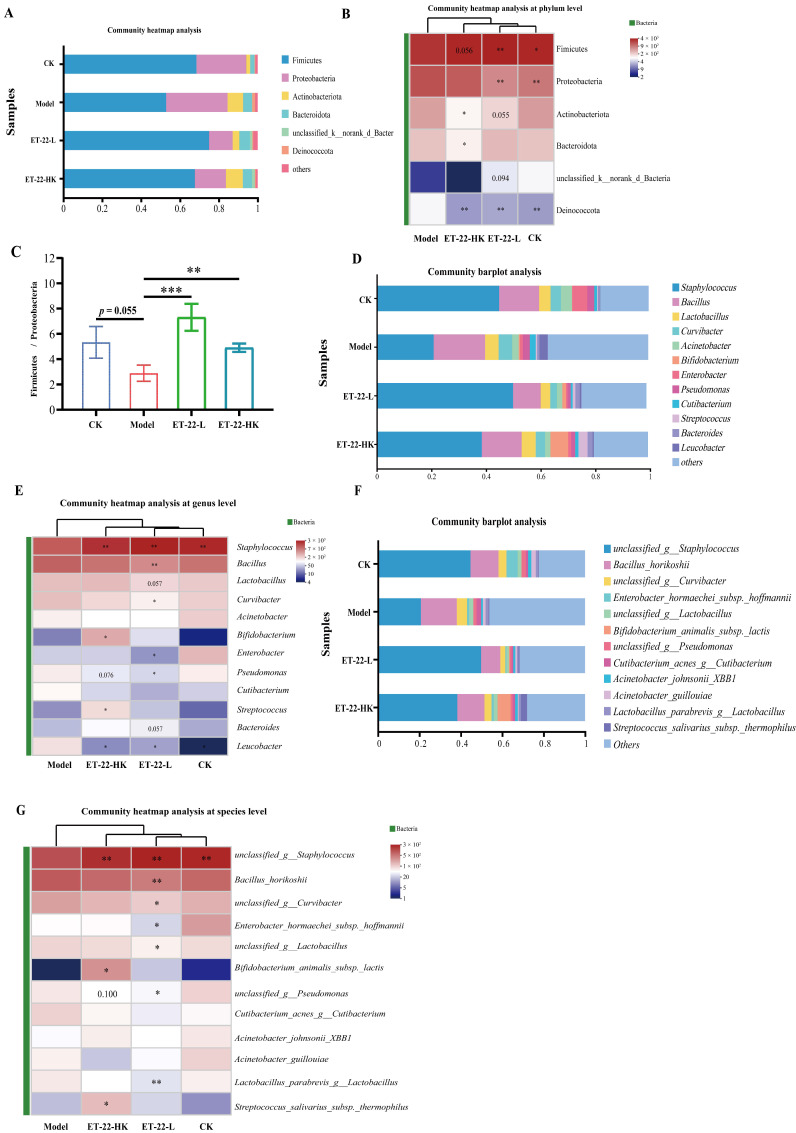
Impacts of ET-22 on microbiota in dental plaques from phylum to species levels. (**A**) The top 6 communities of microbiota in dental plaques at the phylum level. (**B**) The communities changed in relative abundances at the phylum level. (**C**) The changes in the values of Firmicutes/Bacteroidetes. (**D**) The top 12 communities of microbiota in dental plaques at the genus level. (**E**) The communities changed in relative abundances at the genus level. (**F**) The top 12 communities of microbiota in dental plaques at the species level. (**G**) The communities changed in relative abundances at the species level. CK, the mice treated by the sterile water and average diet; Model, the mice treated by the 10% sterile sucrose water and diet 2000; ET-22-L, the mice treated by live ET-22 in 10% sterile sucrose water and diet 2000; ET-22-HK, the mice treated by heat-killed ET-22 in 10% sterile sucrose water and diet 2000. Mean ± SEM are shown (*n* = 10). 0.05 ≤ *p* < 0.1 meant a trend to show significant difference, vs. Model group; * *p* < 0.05, vs. Model group; ** *p* < 0.01, vs. Model group; *** *p* < 0.001, vs. Model group.

In order to identify the communities to play the regulated functions, the relative abundances of communities at different taxonomic levels were compared and shown. At the phylum and genus levels, the top 20 communities with the highest abundances based on the numbers of OUTs were showed in Figure 3A,D. The six most abundant phyla in four groups were Firmicutes, Proteobacteria, Actinobacteriota, Bacteroidota, unclassified_k_norank_d_Bacteria, and Deinococcota, which together covered about 99.05% sequences (Figure 3A). For these six phyla, the relative abundances among four groups were compared and showed in the heatmap (Figure 3B). Compared with the Model group, the CK group presented the obviously reduced abundances of Actinobacteriota, Bacteroidota, and Deinococcota, and showed an increased trend in the abundance of Firmicutes (*p* < 0.05, Figure 3B). Meanwhile, the ET-22-L and ET-22-HK groups all showed significantly increased abundances of Firmicutes and Proteobacteria and reduced abundance of Deinococcota (*p* < 0.05, Figure 3B). The ET-22-L group also showed a decreased trend in the abundances of Actinobacteriota and unclassified_k_norank_d_Bacteria (0.05 < *p* < 0.1, Figure 3B). The value of Firmicutes/Proteobacteria in each sample from four groups was analyzed. Relative to the Model group, the CK group showed a raised trend (0.05 < *p* < 0.1, Figure 3C). The ET-22-L and ET-22-HK groups exhibited obviously increased Firmicutes/Proteobacteria values (*p* < 0.05, Figure 3C). At the genus level, twelve genera with the highest relative abundance were identified in dental plaques, including the *Staphylococcus*, *Bacillus*, *Lactobacillus*, *Curvibacter*, *Acinetobacter*, *Bifidobacterium*, *Enterobacter*, *Pseudomonas*, *Cutibacterium*, *Streptococcus*, *Bacteroides*, and *Leucobacter* (Figure 3D). It is clear that the *Staphylococcus* accounted for the highest proportion in dental plaques of CK, ET-22-L, and ET-22-HK groups, in which the relative abundances of *Staphylococcus* were all higher than the Model group (*p* < 0.05, Figure 3D,E). Meanwhile, the ET-22-L group was higher in the abundances of *Bacillus* and *Curvibacter* and lower in the abundances of *Enterobacter*, *Pseudomonas*, and *Leucobacter* than those in the Model group (*p* < 0.05, Figure 3E). The ET-22-L group also showed a raised trend in the abundance of *Bacteroides* and a reduced trend in the abundance of *Lactobacillus* (0.05 < *p* < 0.1, Figure 3E). In addition, the ET-22-HK group showed higher abundances of *Bifidobacterium* and *Streptococcus* and a lower abundance of *Leucobacter* than those in the Model group (*p* < 0.05, Figure 3E). ET-22-HK group also presented a reduced trend in the abundance of *Pseudomonas* (*p* < 0.05, Figure 3E).

At the species level, multiple communities with high abundances in the dental plaques were observed. The top twelve species were the unclassified_g__*Staphylococcus*, *Bacillus_horikoshii*, unclassified_g__*Curvibacter*, *Enterobacter_hormaechei_subsp._hoffmannii*, unclassified_g__*Lactobacillus*, *Bifidobacterium_animalis_subsp._lactis*, unclassified_g__*Pseudomonas*, *Cutibacterium_acnes_g__Cutibacterium*, *Acinetobacter_johnsonii_XBB1*, *Acinetobacter_guillouiae*, *Lactobacillus_parabrevis_g__Lactobacillus*, and *Streptococcus_salivarius_subsp._thermophilus*, respectively (*p* < 0.05, Figure 3F). Compared with the CK group, the Model group presented a lower abundance of unclassified_g__*Staphylococcus* (*p* < 0.05, Figure 3G). Other species in dental plaques did not make significant changes in the abundances of the Model group (*p* > 0.05, Figure 3G). Compared with the Model group, the ET-22-L group took place great changes in the microbiota of dental plaques and owned a higher abundance of unclassified_g__*Staphylococcus* and lower abundances of *Bacillus_horikoshii*, unclassified_g__*Curvibacter*, *Enterobacter_hormaechei_subsp._hoffmannii*, *unclassified_g__Lactobacillus*, *unclassified_g__Pseudomonas*, and *Lactobacillus_parabrevis_g__Lactobacillus* (*p* < 0.05, Figure 3G). Interestingly, the ET-22-HK group also changed a lot. As shown in Figure 3G, the specie abundances of unclassified_g__*Staphylococcus*, *Bifidobacterium_animalis_subsp._lactis*, and *treptococcus_salivarius_subsp._thermophilus* were higher in the ET-22-HK group.

### 3.4. Functional Predictions of Microbiota in Dental Plaques and the In Vitro Evaluations

**Figure 4:**
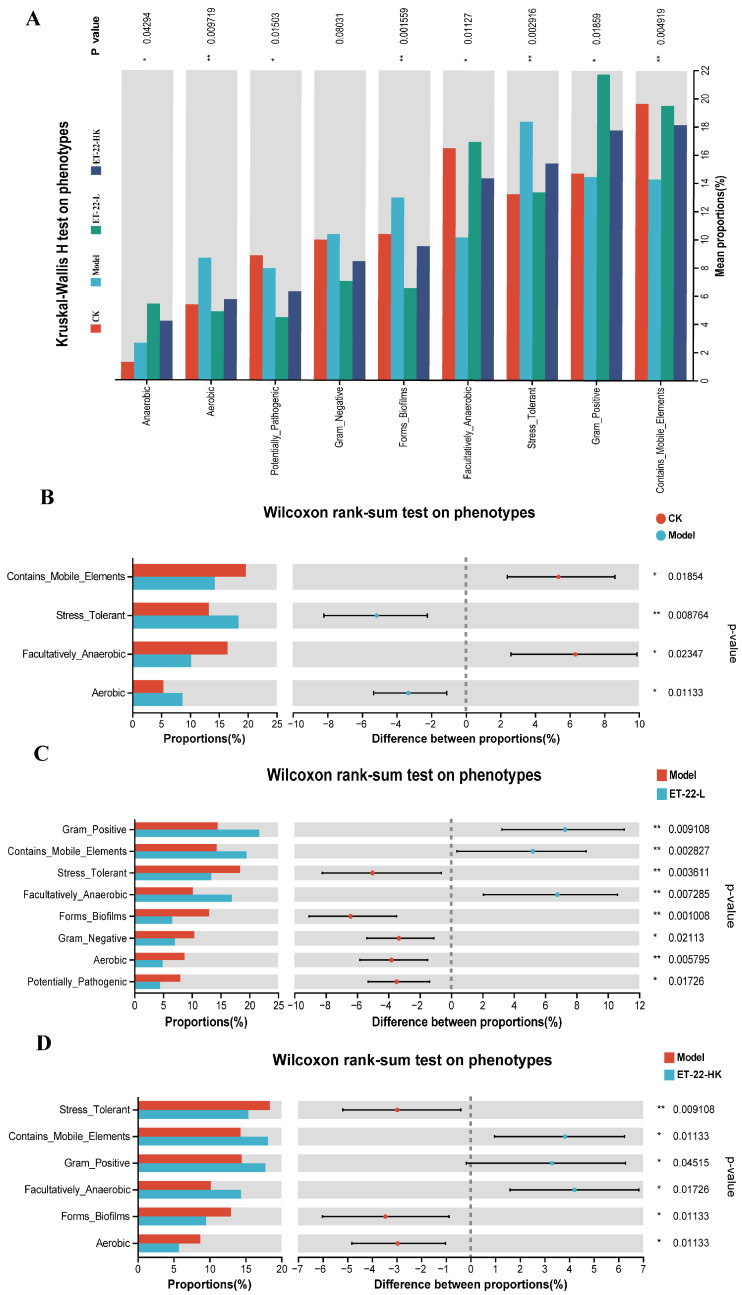
Functional predictions of microbiota by the PICRUSt. (**A**) The Kruskal–Wallis H analysis on the phenotypes among groups. (**B**) The Wilcoxon rank-sum test on phenotypes between CK and Model groups. (**C**) The Wilcoxon rank-sum test on phenotypes between Model and ET-22-L groups. (**D**) The Wilcoxon rank-sum test on phenotypes between Model and ET-22-HK groups. CK, the mice fed with sterile water and average diet; Model, the mice treated by the 10% sterile sucrose water and diet 2000; ET-22-L, the mice treated by live ET-22 in 10% sterile sucrose water and diet 2000; ET-22-HK, the mice treated by heat-killed ET-22 in 10% sterile sucrose water and diet 2000. Mean ± SEM are shown (*n* = 10). 0.05 ≤ *p* < 0.1 meant a trend to show significant difference; * *p* < 0.05; ** *p* < 0.01.

Based on the microbiota in dental plaques, we used the PICRUSt on Majorbio Cloud platform to predict the microbial functions. By the Kruskal–Wallis H analysis, the phenotypes of Anaerobic, Aerobic, Potentially_Pathogenic, Forms_Biofilms, Facultatively_Anaerobic, Stress_Tolerant, Gram_Positive, and Contains_Mobile_Elements of microbiota were predicted to occur differences among groups (*p* < 0.05, Figure 4A). The Gram_Negative phenotype showed a differential tendency among groups (0.05 < *p* < 0.1, Figure 4A). In contrast to the CK group, the Model group was higher in the proportions of Stress_Tolerant and Aerobic phenotypes and lower in the proportions of Contains_Mobile_Elements and Facultatively_Anaerobic phenotypes (*p* < 0.05, Figure 4B). Compared with the Model group, the ET-22-L group was higher in the proportions of Gram_Positive, Contains_Mobile_Elements, and Facultatively_Anaerobic phenotypes, and lower in the proportions of Stress_Tolerant, Forms_Biofilms, Gram_Negative, Aerobic, and Potentially_Pathogenic phenotypes (*p* < 0.05, Figure 4C). Similar to the ET-22-L group, the ET-22-HK group was also higher in the proportions of Gram_Positive, Contains_Mobile_Elements, and Facultatively_Anaerobic phenotypes, and lower in the proportion of Stress_Tolerant, Forms_Biofilms, and Aerobic phenotypes compared to the Model group (*p* < 0.05, Figure 4D).

**Figure 5:**
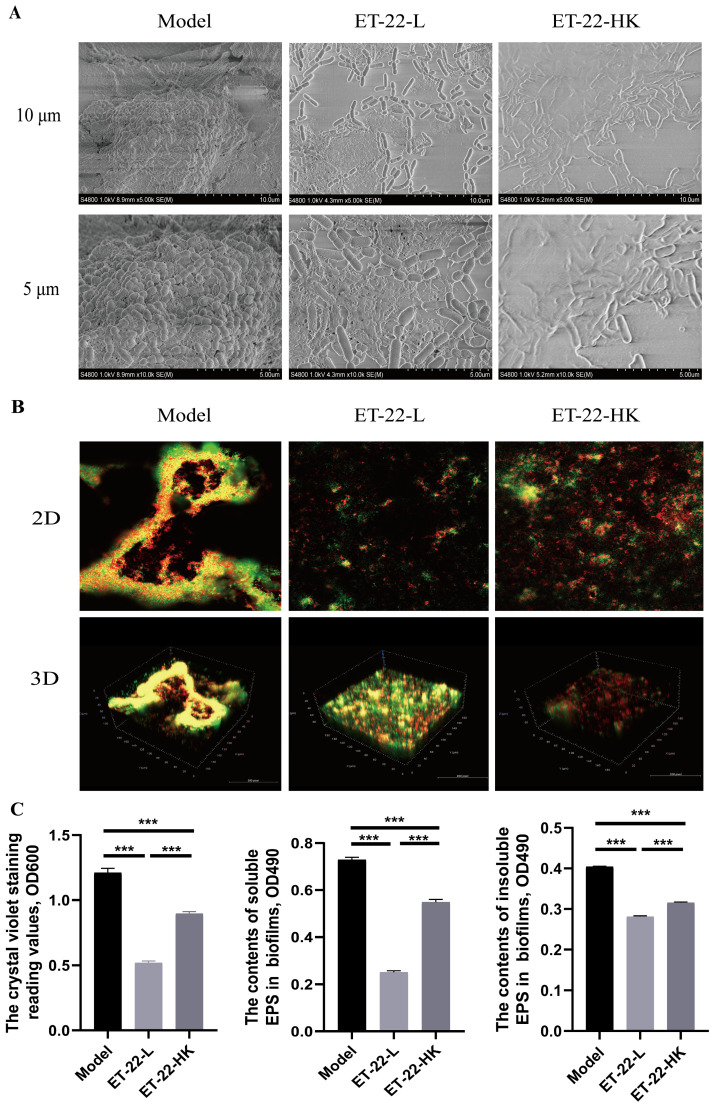
Effect of ET-22 on the formation of plaque biofilms. (**A**) The representative images viewed by the scanning electron microscope, in which the plaque biofilms formed by the *S. mutans*. (**B**) The representative staining images of plaque biofilms viewed by the confocal laser scanning microscopy. Alexa Fluor™ 647 was used to stain the live bacteria and showed the red color. SYTO™ was used to stain the live and dead bacteria and showed the green color. (**C**) Effects of ET-22 on formation levels of biofilms and soluble and insoluble extracellular polysaccharide (EPS) contents in biofilms. Formation levels of biofilms were evaluated by the crystal violet staining reading values under the OD600. The contents of soluble and insoluble EPS in biofilms were checked under the OD490. Model, individual *S. mutans*; ET-22-L, co-culture of live ET-22 and *S. mutans*; ET-22-HK, co-culture of heat-killed ET-22 and *S. mutans*. Mean ± SEM are shown (*n* = 6). *** *p* < 0.001.

In order to verify the functions of microbiota in dental plaques, we used an in vitro biofilm model to check the effect of ET-22 on the plaque biofilms. In the artificial saliva medium added into 1% sucrose, the individual *S. mutans* formed corolliform and compact biofilms on the hydroxyapatite discs (Figure 5A). Compared with the Model group in which the single *S. mutans* was inoculated, the ET-22-L or ET-22-HK groups, inoculated *S. mutans* and live or heat-killed ET-22, showed the no-corolliform and broken biofilm structure (Figure 5A). In the present study, the liver bacteria (yellow) and dead bacteria (green) were shown in 2D and 3D diagrams (Figure 5B). According to the model diagrams, the yellow area accounted for the major proportion (Figure 5B). And, the biofilms in the Model group were coherent and presented a certain height (Figure 5B). However, the yellow area after ET-22-L or ET-22-HK treatment was sparse and mainly in the two-dimensional planes (Figure 5B).

The biofilm formed levels were further detected. By the crystal violet staining, ET-22-L or ET-22-HK treatments significantly reduced the biomass of biofilm compared to the Model group (*p* < 0.001, Figure 5C). Comparison to ET-22-L, the ET-22-HK treatment showed a higher OD600 reading value (*p* < 0.001, Figure 5C). Meanwhile, the contents of soluble and insoluble EPS were checked to verify the reasons for inhibited biofilms. Both different ET-22 interventions significantly inhibited the soluble and insoluble EPS formation compared to the model group (*p* < 0.001, Figure 5C). Similar to the biomass levels of biofilm, the ET-22-HK group showed higher soluble and insoluble EPS contents than the ET-22-L group (*p* < 0.001, Figure 5C).

## 4. Discussion

Oral microbiota dysbiosis seriously damages the health of the oral cavity [8,37]. In order to inhibit the pathogens that disturb oral microbiota, researchers have developed multiple antimicrobial therapies, such as using chlorophyllin–phycocyanin mixture to inhibit *Enterococcus faecalis* [38]. In recent years, probiotics are gaining interest in the prevention and treatment of caries biofilms [39,40,41]. Even though *Lactobacilli* produces organic acid, the overall prevention effect on caries seems beneficial, providing that probiotics candidates are appropriately chosen [42]. According to previous research, *L. paracasei* was one of the *Lactobacillus* with the maximum interference activity against *S. mutans* in vitro [43]. However, whether *L. paracasei* can prevent caries by interfering microecology of dental plaques is unknown. In the present research, an in vivo trial was first conducted to explore the effects of ET-22 on caries-associated microecology.

In this study, the diet with 56% sucrose, 10% (*w*/*v*) sucrose water, and live *S. mutans* was used to prepare the dental caries model. The Keyes caries scoring system including grade Enamel affected (E) and dentin caries (Ds) caries scores is widely used to analyze the severity of dental caries. Based on the increased grade E and Ds caries scores, the dental caries model was successfully constructed in mice of the Model group. Significantly reduced Keyes scores in the ET-22-L group indicate potentially important inhibitory activity of live ET-22 against the progression of dental caries in vivo. In line with this, a double-blind study on Children also found that *L. paracasei* was a candidate used to prevent caries [44]. Similar to the ET-22, a report revealed that *L. plantarum* CCFM8724 showed the best relief of smooth and fissure caries scores, the total score (E) 6.5 and 15.4, respectively [45]. This team also indicated that another strain of *L. plantarum* could reduce the caries scores [5]. This evidence shows that ET-22 may be another strain of *Lactobacilli* to prevent caries induced by *S. mutans*.

In order to investigate the possible mechanism of ET-22, the PacBio full-length 16S rRNA gene sequencing technology was applied to analyze the microbiota of dental plaques. Based on the beta diversity, the administration of *S. mutans* changed the microbiota of dental plaques. Compared with individual *S. mutans* treatment, the live or heat-killed ET-22 also changed the microbiota structure of dental plaques, which was more similar to that of the CK group. According to the Shannon index, the dental caries model induced by *S. mutans* was companied by the increased alpha diversity. The administration of live or heat-killed ET-22 markedly decreased Shannon index, which indicates that ET-22 may play the anti-caries role by reducing the alpha diversity. Combined with previous studies, probiotics administration can maintain the microbial stabilization of dental plaques to defend from the dentes cariosus [46]. This is similar to the chewing of xylitol gum in the mouth cavity in which the cariogenic bacteria were reduced in abundance and species diversity was decreased [47]. The individual study showed that the alpha diversity in the microbiota of plaques from healthy teeth was higher than that from damaged teeth with dental caries [48]. However, some studies found that the microbial abundance and diversity of the oral cavity in children with early caries are not much different from those without [49,50]. The relationship between the microbial diversity of oral or dental plaques and dental caries remains to be further researched.

At the phylum, genus, and species levels, more changes caused by the ET-22 administration could be observed in microbiota of dental plaques. At the phylum level, five of the predominant phyla, including Firmicutes, Proteobacteria, Actinobacteriota, Bacteroidota, unclassified_k__norank_d__Bacteria, and Deinococcota, were largely the same as those previously described for children and adults with dental caries using the pyrosequencing technique [7,48,51,52,53,54]. At the genus level, the communities were mainly dominated by six genera including the *Staphylococcus*, *Bacillus*, *Lactobacillus*, *Curvibacter*, *Acinetobacter*, and *Bifidobacterium*, which was also similar to the previous studies [6,48,54,55,56]. Our study showed that after the treatment of ET-22, the microbiota structure was noticeably different from that of the model group. And supplementation of live or heat-killed ET-22 increased the *Staphylococcus* abundance and decreased the *Leucobacter* abundance. To our knowledge, *Staphylococcus* as the common bacteria associated with dental caries, is not well known in its role in caries initiation and development [57,58,59,60]. According to the phenotype in the presented study, significantly increased *Staphylococcus* in abundance may partly account for the enhanced resistance to dental caries. Decreased abundances of *Bacillus*, *Lactobacillus*, *Curvibacter*, *Enterobacter*, and *Bacteroides* were only shown in mice treated with live ET-22, in which the communities of *Bacillus_horikoshii*, *Enterobacter_hormaechei_subsp._hoffmannii*, and *Lactobacillus_parabrevis_g__Lactobacillus* were specifically inhibited. The metabolites from the live ET-22 may play key roles. It can be speculated that the decrease in caries scoring may result from the above changes in the most extensive species.

An interesting phenomenon is that the heat-killed ET-22 played a similar role in regulating the microbiota of dental plaques to the live ET-22. Butera A. et.al also proved that not only live lactobacilli but also paraprobiotics decreased the percentage of pathological “Red Complex”, and maintained a healthy oral microenvironment [17]. In spite of the different change trends in *Bifidobacterium* and *Streptococcus*, the effects of heat-killed ET-22 on the alpha diversity, beta diversity, and major communities were similar to the live ET-22. This suggests that some functions of ET-22 in regulating the microbiota of dental plagues were performed by the body components of ET-22. In addition, due to the oral environment and presence of lysozyme, the dead ET-22 or ET-22 body may be one of the functional pathways of supplemented live ET-22 in contributing to oral health. Based on the PICRUSt analysis to predict the microbial functions, both ET-22 treatments enhanced the inhibition of biofilm formation. Therefore, the ET-22 body may regulate the microbiota of dental plaques by disturbing biofilm formation. In order to verify this, ET-22 was used to co-culture to check the development of *S. mutans* biofilms. Our in vitro trial suggests that the body components of ET-22 can indeed inhibit *S. mutans* biofilm formation, in which the productions of soluble and insoluble EPS, the elements of the extracellular matrix, were inhibited. Therefore, the inhibition of *S. mutans* biofilms may be another pathway of ET-22 to regulate the microbiota of dental plaques to defend from dental caries. Heat-killed probiotics, called postbiotics, have been found to own important physiological activity in the mucosa [61,62]. Even though the heat-killed ET-22 did not show significant improvement in caries scoring, the long-term supplementation may show a similar preventive effect based on the changes in the microbiota. In future research, it is necessary to investigate the specific functional components of heat-killed ET-22 to prevent dental caries. In addition, whether other strains of *L. salivarius*, *L. casei*, *L. reuteri*, and *L. plantarum* have similar biofilm inhibition functions remains to be confirmed in the future.

## 5. Conclusions

In the present study, we investigated oral administration of ET-22 to view the preventive effect for dental caries of mice inoculated with the pathogenic *S. mutans*. According to the caries scores, the live ET-22 showed the effective defense function. In the further research, we found that ET-22 significantly regulated the structure of microbiota in dental plaques. Heat-killed ET-22, postbiotics of ET-22, play the similar function in regulating microbiota of dental plaques. Based on the functional prediction and in vitro trials, the body compositions of ET-22 were found to inhibit *S. mutans* biofilms by decreasing the EPS productions. Therefore, the ET-22 may be a promising preventative approach for dental caries by regulating structure of microbiota in dental plaques and inhibiting *S. mutans* biofilms. Further studies are needed to evaluate the impacts of ET-22 on microbiota of dental plaques and investigate the concrete functional compositions for biofilms inhibition.

## Data Availability

We permit unrestricted use, distribution, and reproduction in any medium provided the original work is properly cited. Sequencing data generated in this study were deposited in Sequence Read Archive (SRA) (https://www.ncbi.nlm.nih.gov/sra/, accessed on 2 April 2023) with the BioProject ID: PRJNA993434.
